# Supplementary material for: Seaweed and Seaweed Bioactives for Mitigation of Enteric Methane: Challenges and Opportunities
Source: Animals (Basel). 2020 Dec 18;10(12):2432. doi: 10.3390/ani10122432 (PMC7766277; doi:10.3390/ani10122432)
Supplement: Supplementary file 1 [file animals-10-02432-s001.pdf]

Supplementary Table 1: Summary of in vitro studies reporting no methane mitigation effects of seaweeds.

Seaweed species	Substrate	Seaweed dose (% of dry matter (DM) or organic matter (OM) incubated)	Digestibility, TGP or TVFA	Reference
Acrosiphonia spp. (G)	Seaweed alone, spring and autumn	100	Mitigation not examined	[43]
Alaria esculenta (B)	F:C, 50:50	10; harvested spring and autumn	No effect on TVFA	[144]
Alaria esculenta (B)	Seaweed alone (no mitigation), spring and autumn	100	Mitigation not examined	[43]
Ascophyllum nodosum (B)	F:C, 50:50	5	No effect on TGP, TVFA, or DOM (N degradability decreased)	[59]
Caulerpa taxifolia (G)	Rhodes grass hay	2, 3.8, 7.4, 13.8	TGP reduced at dose 7.4:92.6	[50]
Caulerpa taxifolia (G)	Rhodes grass hay	5%	2-5% decrease in TGP, 12% decrease in TVFA, no effect on DOM at 72 h	[11]
Cladophora coelothrix (G)	Flinders grass and CSM	16.6	No effect on TGP	[12]

Cladophora patentiramea (G)	Rhodes grass hay	5%	2-5% decrease in TGP, 23% decrease in TVFA, no effect on DOM at 72 h	[11]
Cladophora rupestris (G)	F:C, 50:50	4.2; harvested spring and autumn	No effect on TVFA	[145]
Cladophora vagabunda (FW)	Flinders grass and CSM	16.6	No effect on TGP	[48]
Cystoseria trinodis (B)	Rhodes grass hay	5%	2-5% decrease in TGP, 12% decrease in TVFA, no effect on DOM at 72 h	[11]
Derbesia tenuissima (G)	Flinders grass and CSM	16.6	No effect on TGP	[48]
Dictyota bartayresii (B)	Rhodes grass hay	5%	2-5% decrease in TGP, 23% decrease in TVFA, no effect on DOM at 72 h	[11]
Ecklonia stolonifera (B) (3 extracts)	Timothy hay	1, 3, 5	No decrease in TGP or DDM; decreased TVFA at 48 h	[77]
Gigartina spp. (R)	Corn silage	25	TGP decreased	[50]
Gracilaria vermiculophylla (R)	Total mixed ration	25	No effect on TGP or OMD	[144]
Halymenia floresii (R)	Flinders grass and CSM	16.6	No effect on TGP	[48]

Laminaria digitata (B)	Seaweed alone (no mitigation),, spring and autumn	100	Mitigation not examined	[42]
Laminaria digitata (B)	F:C, 50:50	5	No effect on TGP, TVFA, or DOM	[58]
Laminaria digitata (B)	F:C, 50:50	10; harvested spring and autumn	No effect on TVFA	[144]
Laminaria ochroleuca (B)	Meadow hay	25	No effect on TGP	[50]
Laminaria ochroleuca (B)	Corn silage	25	TGP decreased	[50]
Mastocarpus stellatus (R)	F:C, 50:50	7.5; harvested spring and autumn	No effect on TVFA	[144]
Mastocarpus stellatus (R)	Seaweed harvested spring and autumn (no mitigation)	100	Mitigation not examined	[42]
Oedogonium spp. (FW)	Rhodes grass hay	2, 3.8, 7.4, 13.8	No effect on TGP	[49]
Padina australis (B)	Rhodes grass hay	5%	2-5% decrease in TGP, 15% decrease in TVFA, no effect on DOM at 72 h	[11]
Palmaria palmata (R)	F:C, 50:50	6.5; harvested spring and autumn	No effect on TVFA	[210]
Palmaria palmata (1 extract) (R)	Timothy grass silage	13, 23, 31	No decrease in TGP or DOM	[9]

Palmaria palmata (2 extracts) (R)	Timothy grass silage	7, 13, 18	No decrease in TGP or DOM	[9]
Palmaria palmata (R)	Seaweed alone, spring and autumn (no mitigation)	100	Mitigation not examined	[42]
Pelvetia canaliculata (B)	F:C, 50:50	7.5; harvested spring and autumn	No effect on TVFA	[145]
Pelvetia canaliculata (B)	Seaweed alone, spring and autumn (no mitigation)	100	Mitigation not examined	[42]
Porphyra spp. (R)	F:C, 50:50	4.2; harvested spring and autumn	No effect on TVFA	[144]
Porphyra spp. (R)	Seaweed alone, spring and autumn (no mitigation)	100	Mitigation not examined	[42]
Saccharina latissimi (4 extracts) (B)	Timothy grass silage	13, 23, 31	No decrease in TGP or DOM	[9]
Saccharina latissimi (B)	F:C, 50:50	7.5; harvested spring and autumn	No effect on TVFA	[145]
Saccharina latissimi (B)	Meadow hay	25	No effect on TGP	[50]
Saccharina latissimi (B)	Corn silage	25	No effect on TGP	[50]
Saccharina latissimi (B)	Total mixed ration	25	No effect on TGP or OMD	[145]
Sargassum flavicans (B)	Rhodes grass hay	5%	2-5% decrease in TGP, 15% decrease in	[11]

			TVFA, no effect on DOM at 72 h	
Spirogyra spp. (FW)	Flinders grass and CSM	16.6	No effect on TGP	[49]
Tarong polyculture (FW)	Rhodes grass	2:, 3.8, 7.4, 13.8	No effect on TGP	[50]
*Ulva ohnoi (*G)	Rhodes grass	5%	2-5% decrease in TGP, 16% decrease in TVFA, no effect on DOM at 72 h	[15]
Ulva rigida (G)	Total mixed ration	25	No effect on TGP or OMD	[145]
Ulva spp. (G)	Corn silage	25	No effect on TGP	[51]

B, brown; CSM, cottonseed meal; DM, dry matter; DOM, degradability of organic matter; F:C, forage:concentrate ratio; FW, fresh water algae; G, green; OM, organic matter; R, red; TGP, total gas production; TVFA, total volatile fatty acids
